# South African single mothers’ experiences of raising a child with a disability

**DOI:** 10.4102/ajod.v12i0.1321

**Published:** 2023-11-17

**Authors:** Siya Mbanjwa, Clare Harvey

**Affiliations:** 1 Department of Psychology, Faculty of Humanities, University of the Witwatersrand, Johannesburg, South Africa

**Keywords:** child, childhood disability, disability, maternal experiences, motherhood experiences, psychosocial support, single mothers, South Africa

## Abstract

**Background:**

Historically, in South Africa (SA), single motherhood has been part of the landscape and continues to increase. Disability in children is also increasing, yet it remains under-researched. Mothers are often left to raise their children with a disability alone, yet their voiced maternal experiences continue to largely be unheard, particularly in SA.

**Objectives:**

This study aimed to explore the lived experiences of single mothers raising a child with a disability in SA. Furthermore, the aim was to explore how these mothers navigate their complex realities and practice of mothering, and to amplify the voices of mothers. Finally, the study sought to shed light on the particular contextual factors that affect single maternal experiences in caring for a child with a disability.

**Method:**

Twelve South African single mothers raising a child with a disability between the ages of 7 years and 18 years were individually interviewed in this exploratory interpretivist study. Thematic analysis was utilised on the data.

**Results:**

The four themes highlight the complex, multi-level strain of raising a child with a disability, which has had a significant impact on the social, financial and emotional facets of single mothers’ lives.

**Conclusion and contribution:**

The findings of the study are important for developing a thorough understanding of the needs of single mothers in this specific context as well as their daily experiences as mothers of children with disabilities. These needs include the necessity of psychosocial support and equipping single mothers with accurate knowledge about their child’s disability so that they can make better accommodations for themselves and their child.

## Introduction

A single mother is ‘a woman who does not live with a partner or spouse and who is the main caregiver for a child under the age of 18’ years, including women who are separated, divorced or widowed (Wright et al. 2013:8). In South Africa (SA), single motherhood can be traced back to the apartheid era; with the migration of men into the cities, women in rural areas were left with the sole responsibility of caring for their children (Moore 2013). Additionally, South African women have traditionally been limited to domestic jobs, emphasising the maternal carer as the social identity of women. Single mothering has become increasingly common both internationally and in SA (Statistics SA 2014). Within divorce custody battles, children are likely to be left in the care of their mothers (Dlamini 2006). Some women choose to raise children on their own to assert their independence (Golombok et al. 2016). Furthermore, even in post-Apartheid SA, absent fathers are a common occurrence (Mkhize 2006).

In SA and worldwide, there is a growing prevalence of childhood disability, yet it remains a relatively under-researched topic, especially in developing countries, including in SA (Yoosefi Lebni et al. 2020). Raising a child with a disability brings about its own unique set of challenges for parents. These challenges are exacerbated by poverty, lack of resources and poor knowledge about disability (Pelchat et al. 2003), factors that may be applicable in low- to middle-income (LMI) SA. The responsibility of caring for a child with a disability typically falls solely on the mother; given the stigmatisation and discrimination of disability, fathers are more likely to neglect their paternal role when their child has a disability (Pelchat et al. 2003). Furthermore, for most men, financial stability is central to practising fatherhood (Kelly 2013), suggesting that the high rates of unemployment in SA may exacerbate the increasing rates of absent fathers.

Parents, particularly mothers of children with disabilities, have rarely been the sole foci in research. Hence, insight into these maternal experiences is limited (O’Connell, O’Halloran & Doody 2013). Much of the literature on child disability appears to be centred on the emotional and caregiving costs on parents, offering overly simple perspectives of parenting children with disabilities (Kearney & Griffin 2001; McConkey et al. 2008). Research focusing on the lived experiences of mothers raising children with a disability has been scarce in LMI countries, including in SA (Mkabile & Swartz 2020; Van der Mark et al. 2019). The study referred to in this article aimed to explore everyday lived experiences and perspectives of South African single mothers raising children with a disability. The focus was on how single mothers of children with a disability navigate their complex realities and practices of mothering, amplifying the voices of these particular single mothers. Furthermore, the study aimed to acknowledge specific contextual factors that influence single maternal experiences in caring for children with a disability in SA.

## Research methods and design

An exploratory, interpretivist qualitative methods approach was used (Pham 2018). Data were collected through in-depth, semi-structured individual interviews online via Zoom or WhatsApp video call, as this was the preferred option indicated by participants. Non-probability purposive sampling was used to access participants (Ritchie, Lewis & Elam 2003). Twelve South African, single mothers with at least one biological child with a disability of school-going age (7 years–18 years) were interviewed. This specific age criterion of the participants’ children ensured that the children are old enough for the mothers to be able to reflect on years of experiences that they have had raising their child. Furthermore, this age range ensured that the insights from the mothers are relatively contemporary as this adds to the richness of the data and contributes to current research. The participants’ children could have any primary disability as the focus of the study was on maternal experiences and less about a particular disability. Lastly, each mother needed to be currently living with their child or at least has lived with their child for most of the child’s life. Please see Table 1 for details of the participants. An advert calling for participants was circulated on the first author’s *Facebook* page and distributed to support groups of parents of children with disabilities. The six steps of thematic analysis (TA) were used on the data (Braun & Clarke 2006, 2023).

**TABLE 1:** Participants’ biographical profile.

Participant	Age of child	Child’s disability
Maria	7 years	Attention Deficit Hyperactivity Disorder ([ADHD]: a behavioural disorder of hyperactivity, impulsivity and inattention [Bowling & Nettleton 2020]). Asperger’s syndrome (neurodevelopmental disorder within the family of autism spectrum disorder; characterised by impaired social communication and interaction [Hosseini & Molla 2022]).
Elizabeth	11 years and 9 years	ADHD Autism spectrum disorder ([ASD]: neurodevelopmental disorder with deficits in social communication and the presence of restricted interests and repetitive behaviours [Hodges, Fealko & Soares 2020]). Language processing disorder (impairment negatively affecting communication through spoken language [Frye 2021]).
Anna	16 years	Cerebral palsy ([CP]: permanent disorders in the development of movement and posture [Sadowska, Sarecka-Hujar & Kopyta 2020]).
Sibongile	16 years	CP
Lindiwe	11years	Physical and mental difficulties, including hyperactivity
Johanna	7 years	CP
Martha	14 years	CP
Busisiwe	14 years	CP
Zanele	8 years	Delayed milestones, difficulties with coordination and speech
Nonhlanhla	8 years	CP
Tebogo	9 years	Difficulties with motor coordination, speech and hearing
Margaret	11-year-old twins	Intellectual developmental disability (impairment of cognitive functions, with limitations of learning, adaptive behaviour and skills [Salvador-Carulla et al. 2011]).

Note: Please see the full reference list of the article, Mbanjwa, S. & Harvey, C., 2023, ‘South African single mothers’ experiences of raising a child with a disability’, *African Journal of Disability* 12(0), a1321. https://doi.org/10.4102/ajod.v12i0.1321, for more information.

CP, cerebral palsy; ADHD,Attention Deficit Hyperactivity Disorder, ASD, autism spectrum disorder.

### Ethical considerations

Ethical clearance was obtained from the University of the Witwatersrand Human Research Ethical Committee (MEDPSYC/21/06). Mothers were presented with an informed consent form before the interview. Written consent was obtained from each participant. No identifying information is revealed here, and each participant has been given a pseudonym to ensure confidentiality and final anonymity.

Raising a child is primarily still seen as a woman’s role with the presumption that mothers will be available to provide care, while the pressure remains for single mothers to be able to maintain a job and be able to meet their child’s needs, often without assistance from the child’s father (Gottlieb 1997). Hence, single mothers take on a variety of demanding responsibilities when parenting their children, and these difficulties are heightened when their child has a disability (O’Connell et al. 2013). In particular, single mothers who lack support and have few resources, such as in LMI contexts, find it particularly difficult and alienating to navigate raising a child with a disability. The overarching theme that emerged from the data is the multi-level strain of single motherhood in the face of child disability. The findings from the study will be presented next (see table 2 for themes from the data).

## Results

### Financial strain

#### Extensive costs

Caring for a child with a disability is expensive. Although an old study, according to Newacheck and McManus (1988), costs involved in raising children with disabilities were two to three times more than those for raising able-bodied children. Furthermore, families in low-income settings have great difficulty in affording the costs of raising a child with a disability, and these costs take up 12% of family income (Leonard et al. 1992; Thrush & Hyder 2014). All participants report expensive disability-related treatment for their children, including surgeries, medications and therapies. In addition to paying for services, participants must travel far to appointments highlighting the lack of and difficulty in accessing resources. As it is too expensive to begin or sustain these, five participants have stopped taking their children for treatments, and others report that their children are on long waiting lists for services:

‘I don’t have the finances to put him on chronic medication. I don’t have the finances to actually go to someone that can really determine what would be the medication for him.’ (Elizabeth, female, single)

‘… I have been trying to get her to wear normal [*shoes*] because her special shoes are expensive.’ (Busisiwe, female, single)

**TABLE 2:** Overarching themes: Multi-level strain of mothering a child with a disability.

Theme	Sub-Theme
Financial strain	Extensive costs Limited finances
It does not take a village	Strain and rejection in romantic relationships Contradictory responses from others
Invisibility of motherhood	‘When your child has a disability, you have a disability’ Advocacy and need for psychosocial support
Survival	Maternal ambivalence to disability diagnosis Maternal knowing, not knowing Resilience

Ten participants’ children do not have medical insurance, which forces many of the mothers to rely on free or low-cost public healthcare. Participants describe the challenges they face in this context, including lengthy wait times, a lack of follow-up care and a general lack of resources and specialists at their disposal. Moreover, participants whose children are on medical aid relay that not all expenses are covered because their children’s treatment is continuous:

‘I have to pay for the medication out of pocket very often …because medical aids are only paying for what they have classified as chronic.’ (Maria, female, single)

‘What she actually needs is to be going to physiotherapy … but going to those at the clinic is hard. It’s been two years waiting for an appointment.’ (Nonhlanhla, female, single)

‘He needs a wheelchair and a walker frame … I’m just busy raising funds for his wheelchair …’cos with the Government I can’t get it …’ (Zanele, female, single)

Arguably, being a single mother of a child with a disability presents particular financial stressors.

#### Limited finances

Compared to families with able-bodied children, children with disabilities and their families are substantially more likely to live in poverty (Shahtahmasebi et al. 2010). The connection between poverty and disability is a significant source of stress for single mothers:

‘… [*O*]ne of the challenges is that if she falls sick, I have to take her to the clinic which costs money. And then from there they refer her to the hospital and then I must ask for money … which is hard.’ (Nonhlanhla, female, single)

‘… [*I*]f I could afford to, I would definitely have had … a psychologist … or I would have definitely had more tests done.’ (Elizabeth, female, single)

Mothers of children with a disability endure a gruelling array of difficulties in caring for their children’s continuing and, at times, overwhelming needs. They have to take on the roles of caregiver, case manager and advocate for their children while navigating a variety of bureaucracies to secure the resources their children need (Parish et al. 2005). Only two mothers receive financial assistance from the fathers of their children, albeit inconsistently. Furthermore, the South African Government Child Disability Grant^1^ is reported to be limited:

‘… [*I*]t’s very difficult getting them to their appointments, then paying for school and their other needs on the little Child Grant we get.’ (Nonhlanhla, female, single)

‘… [*I*]t’s difficult to work and mother a child with a disability … the financial aid is not good enough.’ (Sibongile, female, single)

Given the high costs associated with providing care for children with disabilities, parental employment is essential (Lichter 1997). Factors related to the severity or instability of the child’s disability are strong predictors of reduced hours or cessation of maternal employment (Leiter 2007). Mothers, in particular, do not continue with typical employment trajectories when their children have a disability, and this can have a considerably damaging effect on their family’s financial stability. Eight of the participants here are unemployed, stating that their children’s daily needs prevent them from being able to work full time. Mothers’ abilities to work are hindered by the time commitment required to care for a child with disabilities, as well as a shortage of accessible, affordable childcare (Cuskelly, Pulman & Hayes 1998). Another study found that mothers of children with profound disabilities experience considerable difficulties related to their caregiving responsibilities, including the need to work reduced hours, take leave and switch employment (Shearn & Todd 2001). Similar findings are evident here:

‘… I am only working nightshift. I asked for this when she was born because I couldn’t manage. I didn’t have enough support.’ (Busisiwe, female, single)

‘… I had to not work the whole year to follow up on two operations that he had to get … you need to either … to not work and then suffer the consequences of unemployment or continue working and then your child doesn’t develop.’ (Sibongile, female, single)

‘I can’t [*maintain a fulltime job*] because I must take care of her.’ (Martha, female, single)

‘It was hard [*to keep fulltime employment*] before I found him a school.’ (Lindiwe, female, single)

Finding childcare is a major factor in maternal employment. Concerning childcare for children with disabilities in SA, very little is known. Participants rely on family members and others to help take care of their children; yet, some mothers feel they cannot ‘burden’ these others which prevents many from working:

‘Keeping work is hard because when she comes back from school, she needs someone to care for her. Sometimes they’ll drop her off with the neighbours but eventually even they begin complaining about her, so eventually I had no choice than to just care for her fulltime.’ (Nonhlanhla, female, single)

‘I’ve recently resigned, and I stay home with him … my mum has been taking care of him for the past seven years so I must take over now because he is growing.’ (Zanele, female, single)

Busisiwe made the difficult decision to place her child in a permanent care facility so as to maintain her employment, while others decided to enrol their children in school. However, participants worry about how challenging it is to find suitable and inexpensive education and childcare options:

‘… I have enrolled them in an academy … but it is quite expensive. I had to make quite a few cuts and changes … to ensure that I can afford it.’ (Elizabeth, female, single)

‘… she was [in school … I have to pay school fees for her and transport. That is the challenge and now I am staying with her. She doesn’t go to school.’ (Martha, female, single)

Children with disabilities have continuous demands for specialty care in order to maintain their functioning and health (Perrin 2002). It can be challenging for single mothers to balance the demands of their jobs with the needs of their children. Arguably, participants are left in a paradoxical scenario between choosing to care for their children or being able to support them financially. As a result, many single mothers are forced to rely on government grants and other forms of financial aid that are scarce and insufficient to cover their children’s basic needs.

### It does not take a village

#### Strain and rejection in romantic relationships

Raising a child with a disability causes significant life disruptions, increased levels of distress and marital tension (Sadiki 2023). Unlike the African proverb ‘it takes a village’ to raise a child, the single mothers in this study are largely isolated in their mothering of their children with a disability. The requirement for comprehensive home healthcare and the need for respite are significant when raising a child with a disability, having an adverse effect on parents’ mental and physical health, eliciting feelings of shame, guilt or low self-esteem and drawing the focus away from other aspects of family functioning. All of these stressors can have an impact on the parents’ relationship (Reichman, Corman & Noonan 2008). Parents of children with disabilities are substantially more likely to be separated compared to their counterparts who are raising able-bodied children (McCoyd, Akincigil & Paek 2010). Additionally, having a child with a profound disability increases the likelihood of a separation between the parents, resulting in mothers being left alone to care for their children (Pelchat et al. 2003). According to Pelchat and colleagues, mothers find it difficult to adjust to the needs and demands of their children with a disability, whereas fathers have difficulty adapting to the actual disability. Many participants in this study believe that their partner’s inability to cope with their child’s disability led to their separation. Arguably, the participants’ partners fled when confronted with the knowledge that their children have a disability; the majority of separations occurred during early childhood, when the child’s disability was first diagnosed, and parents were navigating their initial feelings about the disability:

‘… [*A*]round my son’s 5th birthday he came up with an excuse and couldn’t come. I knew he was leaving … he left for good … I no long even consider his dad in our lives … I feel like he doesn’t consider our son to be worth anything and that feels that [*child*] won’t be able to take care of him in his old age which is why he is not investing in him. He only cares for his other children who are well, and he feels will care for him one day.’ (Lindiwe, female, single)

‘… [*W*]hen the second one came along who had more sensory needs … I think in the end it just was too much for him …’ (Elizabeth, female, single)

‘… [*Relationship with father*] very strained. He wasn’t living in the same city as us. He wasn’t supporting him financially … he hadn’t seen my son for about the last two years before he passed.’ (Maria, female, single)

‘… I have a child alone because the father has run away … He left after I gave birth … He doesn’t want the child.’ (Busisiwe, female, single)

‘… [*H*]e’s an absent father. He used to come once in a while … now he’s completely absent.’ (Sibongile, female, single)

‘… [*H*]e was the one who would take her to her check-ups and physio sessions … I think that maybe it became too much for him … When we got the diagnosis it caused me and her father to separate … he’s never come back.’ (Nonhlanhla, female, single)

Eleven participants became single mothers after giving birth to their children with a disability. These results are consistent with those of a European study by Di Giulio, Philipov and Jaschinski (2014), which found 91% of their sample separated after the birth of a child with a disability. The single mothers in this study seem to have a range of emotions to the separation from their child’s father. They all feel abandoned. The belief that their child’s disability was the main factor contributing to their separation brings feelings of bitterness, anger and resentment. The fact is that their partner left an undesirable situation while they could not also elicit these various maternal emotions, including disappointment over the loss of a father figure for their children. As mothers, they do not share the same ‘privilege’ of being able to abandon their children with disabilities.

One of the social ills that SA as a country is currently facing is the status of fathers’ absenteeism and their minimal involvement in the lives of their children (Makusha & Ritcher 2015). According to Richter, Chikovore and Makusha (2010), SA has the second-highest prevalence of father absence, the lowest rate of paternal maintenance for children and the greatest rate of child neglect in the world. Gould and Ward (2015) found that 50% of South African children grow up in homes without their fathers. Families with a child with a disability are particularly vulnerable because there is a considerable danger that fathers reject the child with a disability (Zuurmond et al. 2016). There is compelling evidence that having a father in the home promotes children’s growth, well-being and family functioning (Tracy et al. 2019). Consequently, mothers suffer significant challenges as they are left behind to raise their children by themselves.

Furthermore, only two participants report that their children’s father maintains some level of financial involvement, albeit minimal, in their children’s lives. Thus, most of the participants are left to shoulder the full financial concerns relating to the care of their children, while also being the sole caregiver:

‘He keeps stressing that yah he’s coming but he’s not. He doesn’t fulfil his promises, but I don’t worry much about him. I would be worried if he wasn’t sending money every month.’ (Johanna, female, single)

‘… [*P*]hysically no, he’s just financially there. He’s an ATM^2^ dad …’ (Zanele, female, single)

Fathers are more inclined to disregard their parental responsibilities when their child has a disability because of the stigma and discrimination that accompany it (Pelchat et al. 2003). Furthermore, fathers of children with disabilities appear to associate the mother with the disability too, thus also rejecting her and leaving her to raise their child alone.

#### Contradictory responses from others

Most of the mothers in the study report that they can to various degrees rely on their own family members to help take care of their children:

‘I am also quite fortunate my mother and father … assist me with school pick up and drop off. He spends quite a lot of time with them … I call them co-parents.’ (Maria, female, single)

‘I have two brothers and cousin brothers who are very supportive … with taking him to the toilet … getting him from the transport because he doesn’t come with a wheelchair from school … They just support me.’ (Anna, female, single)

However, some mothers find it difficult to rely on family assistance based on the profoundness of their child’s disability, leaving the single mother largely alone: ‘… they [*family*] say he’s heavy. So they can’t even carry him around … they can’t bath him …’ (Anna, female, single).

Other participants share conflicting responses from their relatives; from taking time to support her to receiving negative feedback regarding her child’s disability, leaving her feeling rejected and alone:

‘… [*W*]hen you have a child with a disability your family rejects you … my mother wants nothing to do with me because I have a child who has a disability [*crying*].’ (Nonhlanhla, female, single)

‘I think the whole thing [*birth of her child with a disability*] was difficult for everyone to adapt to because it’s the first time in my family that we get someone with special needs …’ (Sibongile, female, single)

Ferguson (2002) contends that the sociohistorical environment is inextricably bound up with how a family responds to having a child with a disability. A family’s perception of what it means to be disabled reflects the larger framework of social attitudes and historical circumstances from which that interpretation originates. In the past, many South African tribes viewed disability as a curse from the gods and an omen of doom resulting in harsh, even deadly treatment of the child (Mdziniso 2001; Shabalala 2000). As the mother is the child’s primary carer, this assault and rejection are also directly reflected onto her, making her the target of prejudice and isolation:

‘When my son was first sick [*disabled*] we had tried to consult traditionally … he needed to go to his paternal family. So we went to [*his*] dad who then said that my son was sick because I hadn’t come [*orgasmed*] when I was pregnant.’ (Tebogo, female, single)

Some participants’ families considered alternative explanations, such as them being cursed or that the paternal ancestors had not acknowledged their child, to try to understand why their children have a disability. This brings to light the underlying cultural assumptions and prejudices that prevent families from accepting both the mother and the child with a disability.

According to Goffman (2009), stigma not only impacts the experiences of individuals who hold the stigmatising attribute but also has a propensity to spread to those associated with the individual who holds the negative difference associated with the stigma. Hence, mothers of children with a disability can experience courtesy stigma; their status in society is primarily defined through their children’s disability. The paternal family often believe the mother is responsible for the child and blame her for the child’s disability (Sousa 2011). While the majority of the participants’ ex-partners rejected them and their children after the birth, so too did the paternal families: ‘… no one is involved … there’s no one willing to be involved in her life …’ (Busisiwe, female, single).

### Invisibility of motherhood

‘When your child has a disability, you have a disability’

For some mothers, the arrival of a child with a disability changes their expectations of motherhood and their notions of what is ‘normal’. In a study by Lalvani (2008), some mothers recalled having stereotyped views of people with disabilities and a depressing mental picture of what life would be like for them as the parents of a child with a disability. Prior to the birth of their own children, the majority admit that they had limited contact with people with disabilities. Hence, some mothers find the disability diagnosis to be shockingly life-changing: ‘… it was tough … just hearing that your child has a disability. I thought of the worst case of a disability. It was shocking’ (Anna, female, single).

However, for other mothers, although the diagnosis was difficult to hear, it was a huge relief to finally understand their children’s presentation and be able to help their children: ‘I was literally almost relieved because … everything about who my son is made sense … it was almost a sense of relief but it’s been very, very stressful’ (Maria, female, single). It appears that for mothers like Maria, the relief is followed by worries about how to care for their children and their health, as well as a worry of what would happen to them and how their lives would be forever altered as their mother:

‘… I was relieved ’cause I felt I sort of knew that he was on the spectrum … But then actually realising that he wasn’t ready for school uhm I think it was a bit of a shock. But I think it was a bit of a wakeup call to realise that there is more to just knowing he’s on the autism spectrum.’ (Elizabeth, female, single)

‘… [*I*]t took like ten years just to understand and accept the whole thing. So it was very difficult for years.’ (Sibongile, female, single)

‘… So I was shocked …’ (Johanna, female, single)

The majority of participants appear to have eventually embraced their children’s disability; but despite this acceptance, it is evident how many changes they had undergone and, subsequently, how their meaning of motherhood has changed. Participants shared their hectic schedules that revolve around caring for their children with a disability, resulting in a loss of close relationships and social connections. Hence, having a child with a disability can drastically alter a mother’s life to the point where she feels as if she has a disability as a result of all the changes in her life. For example, Nonhlanhla, a single female, said: ‘when your child has a disability, you have a disability’, and Zanele. also a single female, poignantly stated: ‘I am the disabled one now as a parent with a child with disabilities. He is able to live his own life now but my life has changed’, emphasising the awareness of how their lives change as mothers.

Hence, raising a child with a disability may fundamentally alter how a single mother lives her life because she must constantly attend to her child’s complex requirements. This seems to support the notion that as a single mother of a child with a disability, her life no longer belongs to her and is now centred around providing particular care and safety for her child:

‘… [*W*]hen I wake I do everything for her. I have to bath, make food and then uhm I’m feeding … I do have to change diapers and all those things … It’s very challenging.’ (Maria, female, single)

And, Johanna: ‘I need to prioritise her before anything.’ (Johanna, female, single)

#### Advocacy and the need for psychosocial support

‘As a parent you need to be like pushing systems to work with you … you need to advocate for him … You need to fight for him to get what he deserves’. Sibongile, a single female, illustrates the additional responsibility single mothers have while raising a child with a disability, which entails contending for their children’s care within the systems of care. Mothering goes beyond only providing for the needs of their child and includes advocating for their child (Scott 2010). According to this study’s findings, raising a child with a disability can be physically taxing, expensive and time-consuming. These factors are heightened by a poorly co-ordinated and frequently unresponsive system of service delivery and the fact that single mothers raising a child with a disability also spend a significant amount of time, energy and money on advocacy and other activities.

Notably, not all the mothers encounter hostility from medical professionals and thus do not feel the need to advocate for their children. Many participants have positive experiences in healthcare settings to report, making a significant difference in their ability to care for their children:

‘… [*T*]he physio … The professionals at the school have also been really great.’ (Tebogo, female, single)

‘The relationship with the psychologist is absolutely great …’ (Maria, female, single)

‘… [*T*]he physiotherapist where they explained what kind of a disability he has, eh what normally causes it … they explained a lot of things so I would say I understand better …’ (Anna, female, single)

Professionals who work with single mothers of children with a disability need to establish a foundation of openness, trust and respect. This openness is crucial to support the single mother and help her care for her child despite her many challenges. O’Connell et al. (2013:7) found the value of mothers feeling heard by medical professionals and having others concur that their children could be challenging at times and that they too become stressed. These acknowledgements give mothers the experience of no longer feeling ‘invisible and alone’. This underscores how crucial psychosocial assistance is within the healthcare system:

‘People tend to ask about the child and not ask about us, the mothers.’ (Zanele, female, single)

‘I am a human being as well with my own feelings and emotions.’ (Margaret, female, single)

‘… [*S*]ometimes you need support … counselling as a parent …’ (Martha, female, single)

Some of the participants saw their participation in the interviews in the study as an opportunity to not only speak out on behalf of their children but also share their struggles with other single mothers who had not yet accepted their own child’s disability. Johanna, a single female, mentions that she has helped organise events for parents of children with disabilities. She cites the need for psychosocial assistance among single mothers: ‘there are a lot of people who are still in denial about their children’s problems, therefore we are trying to gather as much as we can so that we can support one other’.

The majority of participants engage in support groups with other mothers of children with disabilities. They speak favourably of the effects of these interactions, stating that it is crucial to have a space where they can be honest about their everyday struggles, particularly at those times when managing their child with a disability was challenging, without fear of being judged:

‘I’m on a WhatsApp parent support group … it’s always nice to be part of something where you can see that everyone is going through the same thing.’ (Maria, female, single)

‘[*Support group*] allows parents to speak up about their challenges. Sometimes you think that what you’re going through is bad until you hear someone else’s story and it makes you cry out of gratitude that things aren’t so bad for you …’ (Nonhlanhla, female, single)

### Survival

#### Maternal ambivalence to disability diagnosis

Eight participants seem to have reached a point of some level of acceptance with regard to their children’s disability. Included in these are an admission of change since the diagnosis, a claim of moving on with life, a suspension of the quest to learn the cause of their child’s disability, an accurate portrayal of their child’s abilities and a balance of claims regarding the benefits of raising a child with this disability:

‘… [*T*]here was a time when I was having a nervous breakdown because everything was so overwhelming. But I feel like I have changed, like I am someone else … overtime I think I have grown to accept. I still have moments when I wonder about my life and where I will end up …’ (Nonhlanhla, female, single)

‘… I realised that it’s not the end. She is just a child. She may be different from other kids but I must accept who she is. I can’t blame anyone. Who was supposed to have a sick child? And then everything fell into place. I accepted the situation.’ (Busisiwe, female, single)

‘It’s just the adapting, to adapt it’s really difficult … I don’t think one will ever be fully content, maybe I’ll get to that level one day, but I’m way better than I was years ago …’ (Sibongile, female, single)

Sibongile seems to suggest that something about the severity of her child’s disability may also play a role in how mothers come to accept their child’s disability. Furthermore, Barnett et al. (2006) found that mothers with the stressors of lower socio-economic positions and/or who identify as minorities, the majority of mothers in SA, have a higher likelihood of being unresolved about their child’s disability diagnosis. The following extract shows how when asked about their early reactions, at least five mothers mentioned financial worries and a lack of awareness about available resources, which made the diagnosis even more confusing and overwhelming:

‘He [*their father*] left me alone with my kids which made things very hard for me. We’ve had to survive on that [*grant*] which is hard because he needs nappies … most of the time she needs soft food. I am a single parent and I don’t work. So we need to use that money for food, clothes, and for her school fees.’ (Nonhlanhla, female, single)

Mothers who have found some resolution with their child’s disability report higher social support and less stress in motherhood compared to mothers with unresolved feelings (Sheeran, Marvin & Pianta 1997). Additionally, Sheeran found that mothers who appear to have advanced in the grieving process related to the trauma of finding out their child’s diagnosis (Harvey 2015) reported that the support they received from other parents, friends and groups to be more helpful than did the group of mothers who were unresolved about their child’s disability. Furthermore, mothers revealed that family support was their preferred coping strategy in the study by Koydemir and Tosun (2009). This was demonstrated in this study, where seven participants state that they relied on their family for assistance when they first learned of their child’s diagnosis. Lindiwe, a single female, explains that she had been so overwhelmed and saddened by the diagnosis to the point that her mother took her child and raised him until she came to terms with his disability: ‘I was in denial and couldn’t understand why my son wasn’t normal. It was very hard for me to understand that and come to terms with it’. And Tebogo:

‘… [*I*]t was very hard for me to accept it. Thankfully my mother was still alive then and asked to take him and care for him. But I struggled a lot. My mom is the one who helped me begin to understand him more.’ (Tebogo, female, single)

In the study by Koydemir and Tosun (2009), mothers claimed that they eventually became accustomed to the circumstances and started to feel more optimistic about their child’s disability. A significant turning point for many of this study’s participants appears to have occurred when they gained more knowledge about their child’s prognosis and clarity about what their child could do and what their demands would be. Evidently, mothers of children with disabilities need to be given reliable information that they can utilise to comprehend their child’s disability and assist their child (Koydemir and Tosun 2009). This is essential in helping mothers accept their child’s disability; it also helps to improve mother–child relationships and gives mothers more capacity to care for their children.

#### Maternal knowing, not knowing

The reactions of shock, frustration and grief to the initial diagnosis of disability in one’s child are often the beginning of the experience of stress for mothers of children with disabilities (Ferguson 2002; Harvey 2015). Following the mothers’ first reactions, they make an effort to comprehend and assess the situation and determine the meaning of the diagnosis in a medical and social context (Lalvani 2008), which can feel both overwhelming and empowering:

‘Once I started … to educate myself on what Asperger’s was … it was almost a sense of relief, but it’s been very, very stressful.’ (Maria, female, single)

‘The doctor explained to me that she struggles to breathe because of something going on in the lungs. I asked what that meant and they began speaking to me about possible developmental delays.’ (Nonhlanhla, female, single)

Most mothers first receive answers to questions about the nature of their child’s disability from doctors, whose explanations, based on the medical model of disability, are often concentrated on the health issues that frequently accompany the diagnosis (Lalvani 2008). However, research by Lalvani revealed that mothers of children with Down’s syndrome are more interested in the social significance of their child’s diagnosis than the medical one. Following the diagnosis of the participants’ children, many mothers worried about the social effects of disability and issues related to what constitutes ‘normalcy’. Arguably, the mothers display fear brought on by not knowing what to anticipate. Sibongile explains:

‘… [*I*]it was very hectic … one minute your child is okay, everything is normal. The next minute they tell you … your child, he’s uh going to live in a different way. It was a very … state of confusion …’ (Sibongile, female, single)

Koydemir and Tosun (2009) explored Turkish mothers’ experiences in raising a child with autism spectrum disorder (ASD). It was discovered that most of the mothers lack accurate knowledge about their child’s disability. These findings are consistent with those of this study, where only nine of the mothers are able to name the disability of their children, and only half of the mothers can articulate their children’s symptoms, demonstrating the knowledge gaps within some mothers. Some mothers, like Anna, can speak confidently about the details of their child’s disability: ‘… now I can sit with someone and explain my child’s condition as if I’m a doctor …’, while Zanele is still confused about her child’s disability: ‘… I am not sure what disability he has … They’ve explained to me that this is how he’ll be his whole life …’

Arguably, some of the mothers feel misinformed because they lack knowledge resources because of limitations in the healthcare system:

‘… [*A*]t the hospital I was told that he’s just going to change the way of living, there was no informative way of them teaching you how to cope with this whole thing. One had to do their own research and learn through time.’ (Sibongile, female, single)

‘The doctor … explained [*child*] had CP [*cerebral palsy*], although no one explained what kind. As time goes on only then did I begin to learn that CP differs. Even now I am not really sure what CP she has …’ (Busisiwe, female, single)

When Elizabeth was asked about what help she wished she had access to, she responded:

‘… [*M*]ore professional services. To be able to really … pin down the diagnoses … to be able to afford those specialists … I don’t have the money to go to a neurologist, but I do wish I could.’ (Elizabeth, female, single)

The difficulty in accessing healthcare and educational systems was identified by Woodgate, Ateah and Secco (2008) as the most worrisome element contributing to parents’ feelings of helplessness and isolation. Manifestations of the inaccessible system include: delaing with professionals who appear to lack specific disability training and knowledge; limited, inadequate and inappropriate resources deemed necessary in order to provide support for raising a child with a disability. Professionals working with children with disabilities need to consider mothers’ feelings, needs and how intimately intertwined their lives are with their children (Doody 2012). It is beneficial for professionals to be especially considerate with mothers and help them fully understand their child’s diagnosis, not only from a medical standpoint but to also provide additional support while they make sense of their child’s diagnosis and their life adjustments as mothers to children with disabilities.

However, the results of this study suggest that not all mothers are receptive to learning about their child’s disability. Not knowing may be a maternal psychological defence mechanism against understanding the demands and challenges their children and themselves face, which can feel unbearable. Lindiwe, in particular, reveals her anxiety about her child’s prognosis and coming to terms with her life changes and expectations of her as the primary caregiver, which was too much to bear. Instead, she remains uninformed and thus able to avoid having to come to terms with what feels like a terrifying reality: ‘… honestly they probably have [*informed her about her child’s diagnosis*] but I have been in denial for years. I was refusing to hear anything they would say at the hospital’.

Contrastingly, several mothers gushed about their understanding of their child’s disability and how over time they had mastered the healthcare system. Maria discussed how she has relied on the knowledge healthcare workers have given her and has supplemented this with her own research to better understand her child and manage her everyday activities. Becoming informed seems to have been a way to feel more in control after the initial shock of learning of their child’s disability.

#### Resilience

Up to now, this study has largely focused on the challenges faced by single mothers of disabled children. However, participants also demonstrate great resilience – the capacity to survive hardship and emerge stronger from it (Heiman 2002):

‘… [*I*]t’s such a relief to talk to someone about mine and my child’s situation because maybe this audio can get to another parent who still hasn’t accepted things.’ (Nonhlanhla, female, single)

‘… I think I’m in a stage whereby I’ve dealt with … most emotional things … I’m kind of content with the condition and how things are in life … it makes you stronger as a person …’ (Sibongile, female, single)

‘Somethings, no matter how difficult they are you must face them because you have no choice.’ (Lindiwe, female, single)

According to Heiman (2002:169), parents raising a child with a disability stress the importance of welcoming their child with a disability, who ‘has the right to live like our other children’, objectively evaluating the situation and attempting to find workable solutions. In this study, Johanna shares how she hosts informative events for parents with children with disabilities, displaying resilience and using her resolve to help herself and other mothers: ‘I really wish to encourage parents of children with disability to … start accepting and taking their children to get help’. Furthermore, Zanele reflects: ‘I don’t want people to say ‘shame’ on me … You have to hold your head high and be proud to be parenting a special child’.

In one way or another, all of the participants have benefited from a variety of support, including extended familial, medical, educational and informal. According to Heiman (2012), informed resilience is demonstrated through asking for help and by taking meaningful action to address one’s circumstances: ‘It’s just very overwhelming to do it on your own … I can’t imagine what people without support uhm how they do it …’ (Maria, female, single)

## Conclusion

This study has highlighted the complex, multi-level strain of single mothers’ lived experiences while raising a child with a disability in SA. Arguably, there is a significant need for psychosocial support interventions to be offered to these mothers that may be accessed in local communities. Further research into community-based non-governmental organisations and services offered by the Department of Social Development is called for. Part of this support should include closing the knowledge gap these mothers have surrounding disability, their children’s needs, prognosis, as well as accommodations for themselves and their children. Other support could come from the children’s fathers, their families and those around the mothers. Consequently, single mothers of children with disabilities will feel less alone and isolated.

## Footnotes

1 In South Africa, the current Child Disability Grant amount is R1980/$109.26 (Government of South Africa 2023).
